# Isolation and Antibiotic Susceptibility of the Microorganisms Isolated from Diabetic Foot Infections in Nemazee Hospital, Southern Iran

**DOI:** 10.1155/2015/328796

**Published:** 2015-12-30

**Authors:** Mojtaba Anvarinejad, Gholamreza Pouladfar, Aziz Japoni, Shahram Bolandparvaz, Zeinab Satiary, Pejman Abbasi, Jalal Mardaneh

**Affiliations:** ^1^Professor Alborzi Clinical Microbiology Research Center, Shiraz University of Medical Sciences, Shiraz, Iran; ^2^Trauma Research Center, Shiraz University of Medical Sciences, Shiraz, Iran; ^3^General Surgery Ward, Nemazee Hospital, Shiraz University of Medical Sciences, Shiraz, Iran; ^4^Department of Microbiology, School of Medicine, Gonabad University of Medical Sciences, Gonabad, Iran

## Abstract

*Background*. Diabetic foot infections (DFIs) are a major public health issue and identification of the microorganisms causing such polymicrobial infections is useful to find out appropriate antibiotic therapy. Meanwhile, many reports have shown antibiotic resistance rising dramatically. In the present study, we sought to determine the prevalence of microorganisms detected on culture in complicated DFIs in hospitalized patients and their antibiotic sensitivity profiles.* Methods*. A cross-sectional study was conducted for a period of 24 months from 2012 to 2014 in Nemazee Hospital, Shiraz, Iran. The demographic and clinical features of the patients were obtained. Antimicrobial susceptibility testing to different agents was carried out using the disc diffusion method.* Results*. During this period, 122 aerobic microorganisms were isolated from DFIs. Among Gram-positive and Gram-negative bacteria,* Staphylococcus* spp. and* E. coli* were the most frequent organisms isolated, respectively. Of the isolates, 91% were multidrug while 78% of* S. aureus* isolates were methicillin resistant. 53% of Gram-negative bacteria were positive for extended-spectrum *β*-lactamase.* Conclusion*. Given the involvement of different microorganisms and emergence of multidrug resistant strains, clinicians are advised to consider culture before initiation of empirical therapy.

## 1. Introduction

Diabetic foot is one of the serious complications associated with diabetes and affects quality of life in respective patients in all ages and races [1]. The World Health Organization (WHO) reported increasing incidence of diabetes all around the world, especially in developing countries. The prevalence of diabetes is on the rise in Iran, chiefly in the southern parts [2, 3].

Neuropathy, peripheral arterial disease, and pressure overload make the sufferers prone to ulcer. It is estimated that approximately 15–25% of diabetic patients develop diabetic foot ulcers during the course of the disease. People with diabetes can progress into chronic ulcers often leading to amputation if not treated promptly [4–6]. Age, male gender, and long duration of diabetes are the other factors associated with amputation [4, 7].

Chronic wounds can be colonized on the surface by a wide range of organisms [8]. Several studies have shown different bacterial agents isolated from patients in different geographical areas in Iran [2, 9, 10]. The inconsistency in reports might be attributed to the varying research methods and populations. If bacterial infection is mild, it is usually monobacterial and if severe infection is present, it is polymicrobial [9]. The antibiotic susceptibility patterns also show variations in diverse geographical regions [11–13].

Multidrug resistant (MDR) bacteria, methicillin resistant* S. aureus* (MRSA), and extended-spectrum *β*-lactamase (ESBL) producing Gram-negative bacteria and their associated complications have created a big health concern among the medical and clinical practitioners [9, 14, 15]. In recent decade, high rates of MDR bacteria, MRSA, and ESBL positive strains have been observed in many hospitalized diabetic foot patients (DFP) [6, 9, 16]. Such conditions make the treatment more demanding and many even menacing to the respective hospitalized patients' lives.

Therefore, early diagnosis of lesions and prompt initiation of appropriate antimicrobial therapy are essential for controlling the infection and preventing complication and improving the quality of life. Antibiotic susceptibility test is a requirement for the management of infections which can help to make better therapeutic choices. Hence, this study was designed to evaluate the prevalence of microorganisms in infected diabetic foot cases and their sensitivity patterns in public hospital, in Fars, Shiraz, Southern Iran.

## 2. Materials and Methods

### 2.1. Patients

This cross-sectional study was carried out on 86 patients admitted with infected diabetic foot. The study design and methodology were approved by the Ethics Committee of Professor Alborzi Clinical Microbiology Research Center and patients' consents were also obtained.

We evaluated the data over a 24-month period from July 2011 to June 2012.

The study population was defined as the total number of patients with Type 1 Diabetes Mellitus (DM) and Type 2 DM with foot ulcers at initial visit and admission to Nemazee Hospital. Information regarding patients' demographic and clinical features such as age, sex, patients' weights, type of diabetes, wound size, random blood sugar level, nature of ulcer based on Wagner classification, and amputation was gathered. Data related to clinical findings such as neuropathy, vasculopathy, nephropathy, hypertension, and retinopathy were also collected.

The vascular disease and neuropathy patients were firstly assessed based on characterization and position of ulcers and their history and then determined with additional tests. For arterial disease, absence of peripheral pulses, presence of claudication, and CT angiography were performed. For neuropathy, nerve dysfunction, significant painful symptoms, reflex test, and light touch sensory were evaluated. Osteomyelitis was diagnosed on suggestive changes in the radiographs and imaging studies.

Diabetes Mellitus was defined according to the criteria set by the WHO [5].

### 2.2. Bacterial Isolation

Two specimens (pus, wound exudates) for microbiological studies were obtained from the infected sites. In fact, the clinical signs of infection and also condition of patients make us use swab culture. For ulcer, the wound before sampling was debrided with a sterile scalpel and rinsed with sterile normal saline and then, samples were collected using sterile swabs, from the depth of the wounds to check for the presence of infective agents (deep swab technique). The swabs were transferred into sterile tubes with brain-heart infusion broth. The tubes were immediately transported to the microbiology laboratory. One swab also was used for Gram staining. The isolates were identified by standard methods. The mold species were identified on the basis of their microscopic and macroscopic appearance.

### 2.3. Antibiotic Susceptibility Patterns

Susceptibility of all the isolates to different antibiotics was determined by the disc diffusion methods, as recommended by the Clinical and Laboratory Standard Institute, using commercial antimicrobial discs (Mast. Co., UK).* E. coli* ATCC 25922 was used for quality control purposes.

MRSA was determined by using the 30 *μ*g cefoxitin disk and oxacillin agar screen plate: the oxacillin (MRSA) agar screen plate was developed for the detection of methicillin resistance in* S. aureus*. Ten microliters of the 10^6^ CFU/mL bacterial suspension (final concentration = 10^4^ CFU/mL) was inoculated onto MHA plates containing 4% NaCl and 6 *μ*g/mL of oxacillin. Any growth occurring within 48 h incubation at 33–35°C was taken to be oxacillin resistant. All* Enterococcus* isolates were examined for reduced vancomycin susceptibility by agar incorporation. ESBL detection for Gram-negative bacilli was done using combined disk method ceftazidime (30 *μ*g), ceftazidime/clavulanic acid (30 *μ*g/10 *μ*g), and cefotaxime (30 *μ*g) and cefotaxime/clavulanic acid (30 *μ*g/10 *μ*g).

All computations for statistical analysis were done by SPSS ver. 19 (IBM, USA).

## 3. Results

### 3.1. Patients' Data

Eighty-six patients with a mean age of 55.5 years hospitalized in surgery wards were studied during the abovementioned period. They included 56 males (65%) and 30 females (35%). The mean duration of diabetes was 13.5 years. DFP weights ranged from 43 to 100 kg, of which 28 cases were over 75 kg (32%). Mean random blood sugar level was 288.5 mg/dL. Median ulcer duration at initial visit was 15 days (range 1–120). Seventy-four (86%) patients received antibiotic treatment on admission (3 patients: cefalexin, 6 patients: imipenem and vancomycin, and 65 patients: clindamycin and ciprofloxacin). Ciprofloxacin 400 mg IV twice a day and clindamycin 900 mg IV three times a day were administered.

Table 1 shows the nature of ulcer and other clinical data. Information about types of ulcers was collected according to the Wagner ulcer classification system. All patients had ulcers graded as 0–4 in the Wagner classification with grade 3 as the most prevalent. Totally, 18 patients presented with osteomyelitis and were diagnosed based on history, physical examination findings, and radiographic and other imaging studies. There were 26 (30%) patients with peripheral arterial disease diagnosed as ischemic ulcer on the day of admission.

### 3.2. Bacterial Isolates and Antibiotic Susceptibility Patterns

Totally, 122 aerobic microorganisms were isolated from the patients. Among them, 78 were Gram-positive, 7 were fungi, and others were Gram-negative.

The most common isolated bacteria were* Staphylococcus* spp. (29%),* Enterococcus* spp. (27%), and* E. coli* (20%). The count and percent of the organisms are presented in Table 2.

**Figure 1:**
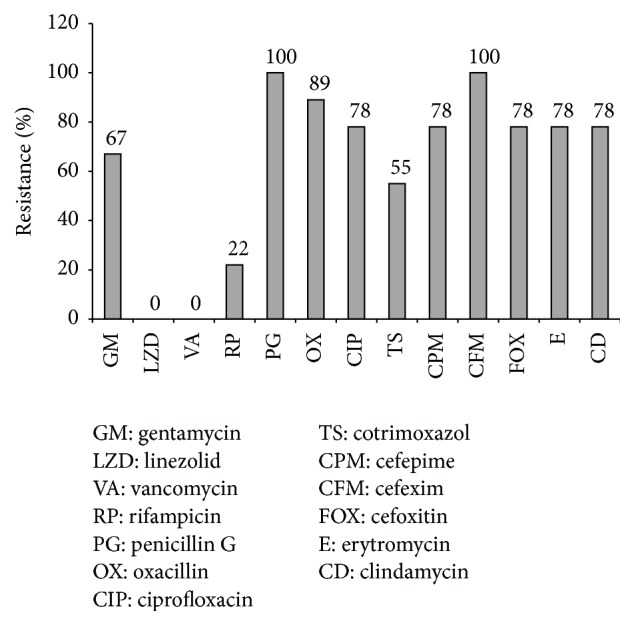
Antibiotic resistance patterns of* S. aureus* isolated from diabetic foot patients.

**Figure 2:**
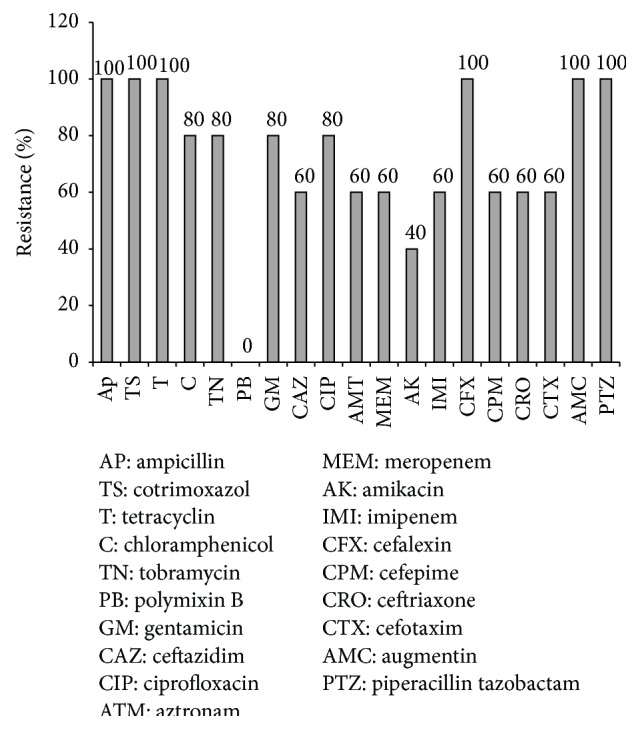
Antibiotic resistance patterns of* nonfermenter* Gram-negative strains isolated from diabetic foot patients.

According to the* in vitro* antibiotic susceptibility testing, linezolid was the most effective antibiotic against* Enterococcus* isolates (all isolates (100%) were sensitive) and ciprofloxacin was the least effective antimicrobial (all isolates (100%) were resistant). As revealed, 20.6% of the* Enterococcus* were resistant to vancomycin. As for* Staphylococcus* spp., linezolid and vancomycin were the most effective antibiotics. 78% of these isolates were MRSA. Resistance rates for* S. aureus* were presented in Figure 1. All Gram-negative bacteria were sensitive to colistin and polymyxin B and 53% of Gram-negative bacteria were ESBL positive. Resistance rates for Enterobacteriaceae and nonfermenters were presented in Table 3 and Figure 2, respectively.

Among 81 bacterial isolates for which we performed antibiotic test, 91% were multidrug resistant; that is, they were resistant to 3 or more antibiotics and considered as MDR [17].

## 4. Discussion

Foot ulceration is the most severe complication affecting diabetic patients which is not confined to certain superficial underlying subcutaneous tissue. Diabetic foot ulceration (DFU) arises from uncontrolled diabetes and incomplete health self-care [18, 19]. This study investigates clinical and microbiological findings of DFU in patients. The majority of the patients with DFU were male and older than 40 years, consistent with other reported studies [7, 20]. This may be due to factors such as the differences in life styles and professional activities and jobs, causing the feet to tolerate more pressure.

In the present study, vascular diseases were the main risk factor among the patients, which is also noted in other studies [21–23]. The percentage of patients involved with neuropathy is comparable to other studies in Iran [24, 25]. Of course, the prevalence varies in different studies, which could be explained by different sample sizes, different distributions of risk factors, and also habits and life styles of the patients such as eating habits and physical activities that can increase the prevalence of risk factors.

The validity and accuracy of using swab versus biopsy culture were addressed in some studies. Some have found that tissue specimens are more sensitive and specific than swab cultures [26, 27] while others have reported that, with debridement, the use of a wound swab, as the most common technique, is as reliable as the use of a tissue specimen, at least for initial monitoring [28–31]. Taking into account the study limitations including the lack of easy access to biopsy samples, clinical signs of infections, and patients' conditions, we were encouraged to use deep swab culture.

In the present study, 79 percent of cases had monomicrobial infection, similar to other studies [32–34], but Lipsky reported in 2012 that polymicrobial strains were most common [35]. The point to be noted here is that we did not consider anaerobic bacteria and monobacterial infections were found to be more prevalent.

We collected 78 Gram-positive bacteria, as in previous studies which reported that Gram-positive strains were more isolated from DFP [9, 10, 34]. In contrast, Gadepalli et al. found that Gram-negative bacteria (*Proteus* species,* E. coli*, and* Pseudomonas aeruginosa*) were predominant strains [6]. Source of infection, use of antibiotic drug for treatment, sample collection method, geographical variation, and type and severity of the infections can influence the pathogens diversity in different geographical areas including Iran. We found that* Staphylococcus* spp. (28 CONs, 9 COPs) were the most frequent pathogens isolated from the patients, as stated in the majority of studies [13, 36].* Enterococcus* spp. were the second most frequent microorganisms which might be due to previous usage of antibiotics. The increased prevalence of* Enterococci* has emerged as a public health concern. Enterococci are frequently detected in compromised patients, such as diabetics, and in their foot ulcers, but their role in infections at these sites is not clearly defined [37].

Gram-negative bacilli and mixed infection were more evident in grades 3 and 4 whereas Gram-positive cocci were most common in grades 1 and 2, indicating that Gram-negative infections increase the severity and make the patients prone to amputation. As revealed, 42 patients underwent amputation, in 32 cases of which Gram-negative bacteria were detected.

Fungal infection was observed in 6% of the patients. In the present study, we isolated 3* C. albicans*, 1* Candida* genus, and 3* Aspergillus* spp. (2* A. niger*, 1* A. flavus*) from cases of DFI. Some studies have noted high prevalence of* Candida* spp. [38, 39], whereas others reported opportunistic molds species as the causative agents of fungal infections in the DFP [38, 40]. Most of the patients involved with fungal infections were in Wagner grades 3 and 4. It is worth mentioning that the patients in our study with diabetic foot ulcers did not receive any antifungal agents at all. So DFP should be examined for fungal infections and also evaluated mycologically for ulcers.

Nowadays, a big concern among the medical and clinical practitioners is the emerging MDR organisms and their associated complications in developing countries [41, 42]. In the current study, ninety-one percent of the bacteria were resistant to three or more antibiotics. Multidrug resistance rates of isolates in 2014 were 63.4%, while the reported rate in 2004 was 18% [43, 44]. To alleviate the rate of MDR and spread of resistance, clinicians should switch to narrower-spectrum therapy.

All the isolates were tested for their antibiotic resistance. The resistance patterns for Enterobacteriaceae, nonfermenters, and* S. aureus* are presented in Table 1 and Figures 1 and 2. The Enterobacteriaceae family was resistant to the majority of antibiotics tested, except colistin, imipenem, amikacin, and meropenem, partially consistent with the results of other studies [11, 45]. But among nonfermenter Gram-negative bacteria, only colistin and polymyxin B were completely sensitive where 60% of strains were resistant to imipenem and meropenem. As for* Staphylococcus* spp., linezolid and vancomycin were the most effective antibiotics and low resistance was seen to rifampicin but a high resistance exhibited to other antibiotics. Other studies have shown different susceptibility antibiotic patterns and approximately in most, vancomycin and linezolid have shown good activity against the strains [9, 46, 47].

The high rates of antibiotic resistance shown in the present study may be due to such factors including hospitalization, recent use of broad-spectrum antibiotics, history of surgery and chronic wounds, irrational use of antibiotics, and the transfer of resistance genes by transport means.

High level of resistance to ciprofloxacin and clindamycin was seen among all the isolates. These antibiotics were used for almost all the patients as empirical therapy in our study. However, it should be noted that clindamycin is also used for anaerobic organisms but we did not examine such organisms in this study. In a study conducted in 2014, it was shown that combination use of these antibiotics does not lead to the desired result [48]. In addition, high rate of resistance to ciprofloxacin was noted by other studies [34, 49].

Although we can not specify an appropriate treatment regimen, considering the results it can be suggested that the best agents for Enterobacteriaceae isolates are polymyxin B, colistin, carbapenem, and amikacin. Polymyxin B and colistin also seem to be the best antibiotics for nonfermenter strains. For Gram-positive cocci, low rate resistance to linezolid and vancomycin was revealed. This could be explained by low prescription of such antibacterial agents for infections in the patients. It seems that these antibiotics can serve as drugs of choice for the treatment of DFI in the abovementioned hospital. On the other hand, consumption of some drugs is limited according to the patients clinical status [36].

As for the alarming types of resistance (i.e., vancomycin resistant enterococci, MRSA, and ESBL), our data showed that the percentages of VRE, MRSA, and ESBL were 20.6%, 78%, and 53%, respectively. These rates are comparable with those in other studies from Iran [9, 13, 50]. Infections with these isolates are more difficult to treat than ordinary ones, because the strains do not respond well to many common antibiotics used to kill bacteria.

To alleviate this situation and also reduce the rate of amputation, clinicians should prescribe antibiotics rationally, timely, and sufficiently and there should be periodic supervisions on the drug consumption by the respective organizations [14].

## 5. Conclusion

It can be concluded that culture specimens for correct management of the DFI and knowledge of the susceptibility of antimicrobial drugs are essential for the choice of appropriate antibiotics with maximum efficacy.

## Figures and Tables

**Table 1:** Demographic and clinical data of diabetic foot patients.

Parameter	Values	[Range or *n* (%)]
Mean duration of diabetes (years)		13.5

Wound size	≤4 mm	22 (25)
	≥4 mm	64 (75)

Weight range	≤50 kgs	4 (5)
	50–75	54 (63)
	≥75	28 (32)

Diabetic type	Type 1	45 (52)
	Type 2	41 (48)

Amputation		42 (49)

Wagner grading of ulcer	0	9 (11)
	I	17 (20)
	II	15 (17)
	III	31 (36)
	IV	14 (16)

Complication	Vascular diseases	48 (56)
	Hypertension	32 (37)
	Neuropathy	22 (25)
	Nephropathy	14 (16)
	Retinopathy	10 (11)

**Table 2:** Frequency of organisms isolated from diabetic foot patients.

Organism	Frequency *N* (%)
*Enterococcus* spp.	34 (27)
*Staphylococcus*: CONs	28 (22)
*E. coli*	25 (20)
*Staphylococcus*: COPs	9 (7)
*Bacillus*	4 (3)
*Candida albicans*	3 (3)
*Proteus* spp.	3 (3)
Diphtheroid spp.	2 (2)
*Pseudomonas aeruginosa*	2 (2)
*Candida* spp.	1 (1)
*Klebsiella pneumoniae*	1 (1)
Beta hemolytic strep. g. A	1 (1)
*Serratia liquefaciens*	1 (1)
*Acinetobacter baumannii*	1 (1)
*Acinetobacter lwoffii*	1 (1)
*Enterobacter gergoviae*	1 (1)
*Morganella morganii*	1 (1)
*S. maltophilia*	1 (1)
Fungi	3 (3)
Totally	122

**Table 3:** Antibiotic resistance patterns of Enterobacteriaceae (32) strains isolated from diabetic foot patients.

Antibiotic	Total resistant strains *N* (%)
Tetracycline	29 (90)
Ampicillin	27 (84)
Trimethoprim-sulfamethoxazole	25 (78)
Ciprofloxacin	25 (78)
Amoxicillin clavulanic acid	21 (65)
Cefalexin	21 (65)
Cefotaxime	18 (56)
Chloramphenicol	18 (56)
Tobramycin	18 (56)
Aztreonam	17 (53)
Ceftriaxone	17 (53)
Ceftazidime	16 (50)
Cefepime	15 (47)
Piperacillin tazobactam	13 (40)
Gentamicin	13 (40)
Meropenem	3 (9)
Amikacin	2 (6)
Imipenem	1 (3)
*Polymyxin B, colistin*	*0 (0)*
